# Associations between Sleep Characteristics and Cardiovascular Risk Factors in Adolescents Living with Type 1 Diabetes

**DOI:** 10.3390/jcm11185295

**Published:** 2022-09-08

**Authors:** Nana Wu, Veronica K. Jamnik, Michael S. Koehle, Yanfei Guan, Yongfeng Li, Kai Kaufman, Darren E. R. Warburton

**Affiliations:** 1 Physical Activity Promotion and Chronic Disease Prevention Unit, The University of British Columbia, Vancouver, BC V6T 1Z4, Canada; 2 School of Kinesiology and Health Science, York University, Toronto, ON M3J 1P3, Canada; 3 Division of Sports Medicine, Faculty of Medicine, The University of British Columbia, Vancouver, BC V6T 1Z4, Canada; 4 School of Kinesiology, Faculty of Education, The University of British Columbia, Vancouver, BC V6T 1Z4, Canada; 5 College of Sports and Health, Shandong Sport University, Jinan 250102, China

**Keywords:** cardiovascular health, sleep, type 1 diabetes

## Abstract

Adolescents living with type 1 diabetes (T1D) have an increased risk of developing cardiovascular disease. Sleep patterns have physiological and behavioral impacts on diabetes outcomes. This study aimed to investigate the associations between sleep patterns and CVD risk factors in adolescents living with T1D and their peers living without T1D. This cross-sectional study assessed CVD risk factors and sleep characteristics (and their associations) in adolescents, aged 12–18 years, living with T1D (*n* = 48) and their peers (*n* = 19) without T1D. Outcomes included blood pressure, lipid profiles, and sleep characteristics (accelerometry). Statistical differences between groups were determined with chi-square or independent samples *t*-tests. The associations between sleep characteristics and CVD risk factors were assessed with multivariate linear regression analyses. We found no significant differences between the two groups in terms of sleep duration, efficiency, sleep onset and offset, and frequency of awakenings, and there were associations between sleep efficiency and LDL-C (β = −0.045, *p* = 0.018, model R^2^ = 0.230) and triglycerides (β = −0.027, *p* = 0.012, model R^2^ = 0.222) after adjusting confounders (diabetes status, sex, age, pubertal stage) in all participants. In conclusion, adolescents with T1D and without T1D sleep less than the recommended eight hours per night. The associations between sleep efficiency and LDL-C and triglycerides are independent of sleep duration, regardless of sex, age, and pubertal stage.

## 1. Introduction

Cardiovascular disease (CVD) is the most prevalent cause of premature death and disability in individuals living with type 1 diabetes (T1D). Cardiovascular risk factors associated with T1D can develop in childhood and adolescence [1]. Previous research has revealed that 76% of children and adolescents with T1D were found to have one or more risk factors for CVD (i.e., obesity, hypertension, hyperglycemia, or dyslipidemia) [2]. In fact, the childhood onset of T1D has been associated with a higher risk of developing CVD in adulthood [3].

Short or very long durations of sleep and/or poor sleep quality in childhood are associated with a higher risk of CVD in adulthood [4], and may negatively impact the child’s long-term cardiovascular health [5]. Moreover, poor sleep patterns are related to CVD morbidity and mortality in adulthood [6]. Previous research has shown that acute changes in CVD risk factors, including blood pressure, heart rate, glucose and insulin metabolic indices, and inflammation, often occur when healthy participants are deprived of sleep for varying lengths of time [7,8].

Emerging evidence has compared sleep duration among youths living with T1D versus youths without T1D and has identified some degree of disturbed sleep characterized by a reduction in sleep duration [9,10,11,12]. This includes an increase in overnight awakenings [13,14], alterations in sleep architecture [10,11], and poor sleep quality [11]. However, most of these studies are based on subjective self-reporting (often known to inaccurately estimate both sleep quantity and quality) and many include small sample sizes [15]. Moreover, sleep problems and disruption can affect insulin sensitivity and glucose regulation [16,17]. Currently, there are limited studies that investigate how sleep quality affects cardiovascular risk in adolescents living with T1D. Sleep has physiological and behavioral impacts on diabetes outcomes; however, little is known about the impact of sleep disturbances on CVD risk factors in adolescents with T1D.

Wrist accelerometry is a movement-based, non-invasive, and relatively low-cost approach to estimating sleep patterns. The potential benefits of using accelerometry, as acknowledged by the American Academy of Sleep Medicine, are as follows [18]: first, data can be collected over multiple days and nights at home and are more natural and representative of the individual’s usual habits. Second, it is relatively affordable, minimally invasive, and easy to complete for most people. In comparison, polysomnography measures sleep stages based on brain activity and biological parameters (e.g., breathing and oxygen levels) and is generally considered as the gold standard of sleep measurement; however, it can only be performed in a laboratory and individuals may sleep differently in a laboratory than at home, which can affect the results. In addition, it is difficult for adolescents living with T1D who are required to manage nocturnal hyperglycemia or hypoglycemia. Moreover, previous studies have demonstrated that accelerometry, taken simultaneously with polysomnography, is valid and reliable in measuring the duration of sleep and sleep efficiency in healthy individuals [19,20,21]. Therefore, the use of accelerometry for the assessment of sleep patterns is warranted when working with adolescents living with T1D.

Accordingly, the primary objective of this study was to compare objectively measured sleep patterns (accelerometry) in adolescents living with T1D and with peers living without T1D. The second objective was to investigate the relationship between sleep patterns and CVD risk factors. We hypothesized that there would be an increase in sleep disturbances in adolescents living with T1D when compared to adolescents living without T1D, and poor sleep quality and a shorter duration of sleep would be associated with proatherogenic CVD risk factors.

## 2. Materials and Methods

### 2.1. Participants

**Figure 1:**
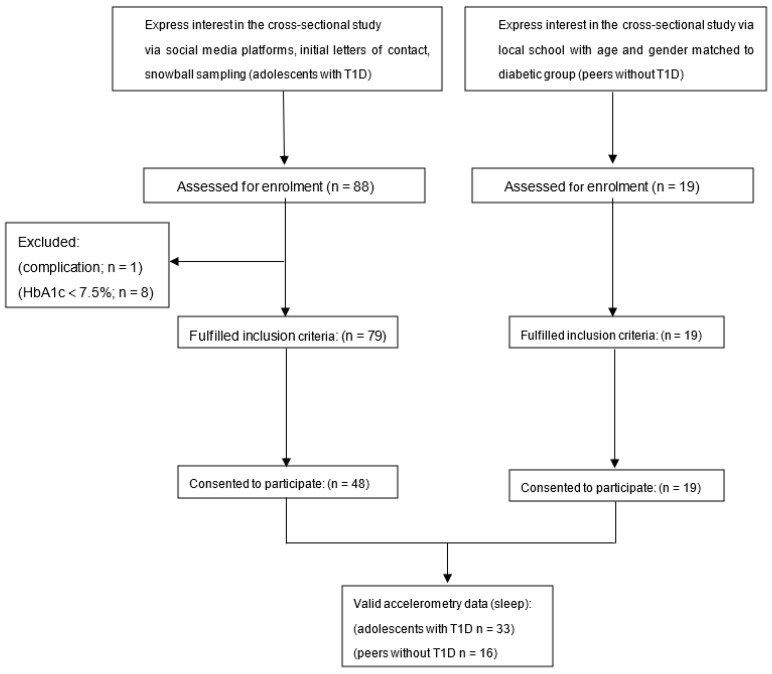
Flowchart of participant screening.

A cross-sectional study of 48 adolescents living with T1D (the World Health Organization (WHO) criteria) and 19 apparently healthy peers living without diabetes (aged 12 to 17 years) was conducted. Figure 1 shows the flow of participant screening. We completed the recruitment of adolescents with T1D via advertisements using social media platforms (e.g., Diabetes WeChat groups), by distributing initial letters of contact to individuals with T1D, and snowball sampling. The inclusion criteria for individuals living with T1D were: (1) at least 6 months of diagnosis of T1D; (2) with HbA1c greater than or equal to 7.5% in the last three months; (3) with normal renal function; and (4) free from previous CVD and chronic kidney disease. The exclusion criteria of participants were: (1) with any significant diabetic complications (diabetic foot, retinopathy, severe neuropathy); (2) with uncontrolled hypertension; (3) with diabetic ketoacidosis; (4) with CVD (defined as any form of clinical coronary heart disease, stroke, or peripheral vascular disease); or (5) with severe hypoglycemia episodes within the past 3 months.

We recruited peers without diabetes to match for age and sex to the group living with T1D from local schools via snowball sampling. The included healthy peers had no known history of chronic disease and no clinical or laboratory evidence of CVD, or other pre-existing health conditions that would contraindicate/limit their participation in regular physical activity. Healthy participants that required the use of any medications, which may affect lipid profiles, cardiovascular function, and/or glucose metabolism, were excluded from the study. This study was part of a project that has been published previously [22]. In total, 67 were included in this study; a total of 48 participants living with T1D and 19 participants were peers living without diabetes.

### 2.2. Demographic and Anthropometric Data Collection

We measured height in bare feet to the nearest 0.1 cm with a wall-mounted stadiometer, and body weight was measured to the nearest 0.1 kg in light clothing utilizing a BC-418 segmental body composition analyzer (Tanita, Tokyo, Japan). We calculated body mass index (BMI) as weight (kg) divided by height squared (m^2^). In order to assess puberty stages, a validated self-report questionnaire with images of pubertal staging was utilized, and the adolescents were categorized as pre-pubertal (Tanner 1), in early puberty (Tanner 2), mid-puberty (Tanner 3–4), or post-puberty (Tanner 5) [23]. This questionnaire was validated for Chinese children [24]. Participants living with T1D were required to complete questionnaires upon arrival at the testing location in order to determine age, sex, diabetes history, duration of diabetes since diagnosis, complications, medications, and insulin regimen. Peers living without T1D were asked to complete a questionnaire about their medical history as well. We conducted waist circumference measurements in triplicate using a flexible tape at the midpoint between the top of the iliac crest and the lower margin of the last palpable rib [25]. We measured resting blood pressure after 10 min of rest in the seated position and the average (three measurements taken one minute apart) was used in the analysis.

### 2.3. Cardiovascular Outcomes

Participants’ body compositions were obtained from dual-energy X-ray absorptiometry (DXA) using the iDXA instrument (GE Medical Systems, Madison, WI, USA) with Encore 2011 software (version 13.6). On the days when data were collected, all participants had fasted overnight for at least 12 h and the T1D group did not administer their morning insulin injections. We took fasting capillary (fingertip) blood samples for analysis of HDL-C, LDL-C, total cholesterol, and triglycerides, and these lipid profiles were analyzed using the Cardiochek PA Blood Analyser (Polymer Technology Systems Inc., Indianapolis, IN, USA). The correlation between the CardioChek PA Blood Analyzer from fingerstick blood samples and venous blood samples was high for HDL-C (r = 0.95), triglycerides (r = 0.95), and total cholesterol (r = 0.88) [26]. In addition, we used the A1cNow+ (Metrika Inc., Sunnyvale, CA, USA) to analyze HbA1c from a fingerstick blood sample, which has been demonstrated to be highly correlated with the high-performance lipid chromatography test of using a venous blood sample (r = 0.95) [27]. These devices have been previously validated as well [28,29].

### 2.4. Accelerometry Assessment of Composition of the Day

We used a 7-day sleep diary and wrist accelerometer (wGT3x-BT ActiGraph LLC., Pensacola, FL, USA) to collect information on sleep. Participants were asked to wear the accelerometers on their non-dominant wrist for seven consecutive days (five weekdays), except in the water, as the device is water-resistant, but not waterproof. Accelerometry has been reported as a valid and reliable method to assess sleep parameters (intraclass correlation coefficients, ICC = 0.63–0.81) [30]. Sleep data were analyzed using the manufacturer’s software (ActiLife Version 6) with the Sandeh sleeping scoring algorithm [31]. This algorithm determines a participant’s sleep state by examining the activity counts over an 11 min window. Probability analysis is used to define each minute of recorded activity (using an 11 min sliding window) as either an awake or sleep epoch by weighting the activity scores of the surrounding minutes. If the probability is zero, the specific epoch is scored as sleep; otherwise, it is scored as awake [31]. At least four nights of wear time (three weekdays and one weekend) were required to be considered valid; a total of 33 individuals living T1D and 16 peers living without T1D had valid sleep data for analysis.

In order to objectively measure sleep, we included the following variables: estimates of sleep timing (sleep onset and sleep offset), total sleep time (time between falling asleep and final awakening, from which the time spent awake in between is subtracted), sleep-onset latency (time between lying down in bed and falling asleep), sleep efficiency (total sleep time divided by total time in bed, in %), number of awakenings (the number of different awakening episodes as scored by the algorithm), wake after sleep onset (time awake between falling asleep and final awakening), and length of awakenings (the average length of all awakening episodes). An awakening does not necessarily mean the participant is awake, but rather that there was enough movement within the epoch (minute) to mark that epoch as “awake”).

### 2.5. Statistical Analyses

All statistical analyses were conducted using SPSS 20 for Windows (Statistical Package for the Social Sciences, IBM Corp., Armonk, NY, USA) and data were screened for normal distribution. The chi-square test was used for comparison of proportion (categorical variables including sex and pubertal status) between groups, and independent samples *t*-tests were used for comparisons of the continuous variables (age, height, body mass, body composition, BMI, and sleep variables) between groups. A non-parametric test (Mann–Whitney) was used to compare sleep onset and sleep offset among groups. Results were summarized using medians and 25–75th quartile for non-normally distributed variables, using frequencies and percentages for categorical variables, and means and standard deviation (SD) for normally distributed variables. These associations between sleep characteristics and CVD risk factors were then assessed with univariate linear regression analysis (Step 1) in the T1D group and apparently healthy peers without diabetes. The multivariate linear regression analysis (Step 2) was adjusted for age, sex, and pubertal stage in individuals with T1D and in apparently healthy peers not living with diabetes. The *p* value less than 0.05 was considered to be statistically significant.

## 3. Results

The demographic characteristics of participants with T1D and peers without diabetes are displayed in Table 1. No significant differences were found in age, sex, pubertal stage, height, body mass, body fat percentage, BMI, waist circumference, or blood pressure. T1D participants showed significantly higher values of total cholesterol (4.03 ± 0.81 vs. 3.14 ± 0.67 mmol·L^−1^, *p* = 0.001), LDL-C (2.31 ± 0.72 vs. 1.74 ± 0.38 mmol·L^−1^, *p* = 0.035) and triglycerides (0.89 ± 0.31 vs. 0.60 ± 0.40 mmol·L^−1^, *p* = 0.012) compared to peers without diabetes.

There were no significant differences in sleep characteristics between adolescents living with T1D and without T1D (Table 2). We investigated the within-group relationships between CVD risk factors (blood pressure, HbA1c, HDL-C, LDL-C, total cholesterol, and triglycerides) and sleep parameters with bivariate analysis. In adolescents living with T1D, no significant associations were found between CVD risk factors and sleep characteristics. In healthy peers without T1D, the LDL-C and triglycerides both correlated negatively with sleep efficiency (r = −0.554, *p* = 0.026 and r = −0.617, *p* = 0.011) and no significant associations were found between any other CVD risk factors and sleep characteristics.

Linear regression models are presented in Table 3 and showed that LDL-C in adolescents without T1D was negatively associated with sleep efficiency (β = −0.0554, *p* = 0.026, model R^2^ = 0.307). However, the results changed to a negative relation trend after adjusting for age, sex, and pubertal stage (β = −0.04, *p* = 0.071, model R^2^ = 0.395), with a stronger relationship.

In the entire sample size (*n* = 49), we performed multivariate linear regression analysis including CVD risk factors as dependent variables, and diabetes, age, pubertal stage, and sleep characteristics (sleep efficiency and sleep duration) as independent variables. We found sleep efficiency was independently associated with LDL-C (β = −0.045, *p* = 0.018, model R^2^ = 0.230) and triglycerides (β = −0.027, *p* = 0.012, model R^2^ = 0.222) (Table 4).

## 4. Discussion

On average, adolescents living with T1D and without T1D in our study slept less than the recommended eight hours per night. A significant correlation between sleep efficiency and LDL-C and triglycerides was observed when confounders of age, sex, and pubertal stage were adjusted in healthy peers without T1D and in the entire sample adjusted for diabetes, age, sex, and pubertal stage. Similar to the findings in previous research, our study did not find any significant differences in sleep duration [10], efficiency [32,33], sleep onset and offset, or frequency of awakenings [32] between individuals living with T1D and their peers living without T1D.

Our study found no significant differences in sleep duration between adolescents living with T1D and peers without T1D. Similar results have been demonstrated by Perfect et al. [10] and Macaulay et al. [33]. However, a systematic review and meta-analysis of sleep characteristics and associations with glycemic control in T1D showed that children and adolescents slept an average of 26 min (by objective measurement) less than peers without diabetes. The reasons for the inconsistency of these results are unclear. Interpretations of these findings should be considered with caution. There were only three small studies with a total sample size of 70 participants living with T1D. Furthermore, strict inclusion criteria of participants without chronic complications were used [11]. Manin et al. reported that the prevalence of obstructive sleep apnea was high among males living with T1D [34]. In addition, obstructive sleep apnea was independently associated with macrovascular complications and retinopathy [34]. Our study excluded participants living with chronic complications, which may explain the similarities between the adolescents living with T1D and their peers living without T1D. Moreover, subjective sleep data were not available, and sleep time was based on sustained bouts of inactivity during sleep (11 min window) within a predefined, angular range of arm motion, which may lead to a potential underestimation of total sleep time [35].

The latest guidelines, based on available evidence of the impact of sleep duration on health outcomes, published in 2016 by the American Academy of Sleep Medicine, recommended that children (aged 6–12 years) should obtain 9–12 h of sleep per 24 h [36]. Moreover, adolescents (aged 13–18 years) should obtain 8–10 h of sleep per 24 h [36], and adults should obtain 7 or more hours of sleep per 24 h [37]. In our study, neither the adolescents living with T1D group nor without T1D group achieved the minimal recommended time of daily sleep (8 h). We found that individuals with T1D slept 58.7 min and peers without T1D slept 47.98 min less than these recommendations. Previous research has reported that insufficient sleep durations in Chinse adolescents are due to heavy academic burdens [38]. A growing body of evidence reported similar results in American [39], Canadian [40], German [41], and Australian [42] children and adolescents, and insufficient sleep has been identified as an international public health concern.

Evidence suggests that inadequate sleep quality and quantity are causally linked to sleepiness, inattention, cognitive and behavioral deficits, and long-term functional development [43]. Previous cross-sectional and longitudinal studies have established associations between inadequate sleep and increased prevalence of overweight and obesity among children and adolescents [44,45]. Furthermore, previous studies have linked a shorter duration of sleep to metabolic dysfunction in children and adolescents [46,47,48,49]. Spruyt et al. found that a shorter sleep duration was associated with obesity and poorer metabolic health, including glucose, insulin, cholesterol, triglycerides, and high-sensitivity C-reactive protein in children aged 4–10 years old [46]. These findings highlight the importance for children and adolescents to obtain an adequate amount of quality sleep on a regular basis.

We found no significant association between sleep duration and glycemic control in adolescents living with T1D who had no reported CVD complications. Similarly, Perfect et al. found that objectively measured sleep duration was not related to glycemic control in adolescents living with T1D [10]. This finding is also supported by Reutrakul et al.’s meta-analysis [11]. In contrast, findings in Reutrakul et al.’s systematic review highlighted that shorter self-reported sleep duration and poorer self-reported sleep quality was associated with increased HbA1c, with a mean difference in HbA1c of 0.19% and 0.24%, respectively, in adults with T1D [11]. On the other hand, Hazen and colleagues found that, in addition to poorer glycemic control and higher average blood glucose levels, parent reports of their children sleeping longer than other children were associated with poorer T1D management [50]. Collectively, these findings suggest that obtaining sleep outside the recommended range (whether too little or too much) may have a negative impact on glycemic control for individuals with T1D. The optimal and minimal amounts of sleep duration and quality that yield positive and beneficial changes in the cardiovascular risk profile of those living with T1D remain to be determined.

A significant inverse relation between sleep efficiency and LDL-C was found in healthy peers without T1D, and the trend for an inverse relation remained after adjusting for sex, age, and pubertal stage. However, the clinically relevant need is still to be determined as these beta coefficients were small. In addition, we found no significant association between sleep efficiency and HbA1c and other CVD risk factors in adolescents living with T1D. In contrast, von Schnurbein et al. reported that self-reported increases in average sleep quality were correlated with a small decrease in HbA1c among individuals living with T1D [51]. The lack of correlation in our study is likely due to potential confounders of insulin therapy modality and diabetes management, excluding individuals with T1D who had diabetic complications and a small sample size. With multiple regression analysis, the association between sleep efficiency and LDL-C and triglycerides is independent of sleep duration after adjusting for confounders (diabetes, sex, age, and pubertal stage) in our study. Similarly, Narang et al. found poor sleep quality was independently associated with cardiovascular risk factor abnormalities in healthy adolescents [49]. Additionally, Jarrin et al. found that sleep quality was negatively linked with obesity in children and adolescents [52]. These observations highlight the importance of optimizing sleep quality and sleep duration for the management of T1D and increasing the awareness of early treatment of CVD risk factors in youth with T1D.

### Strengths and Limitations

To our knowledge, this is the first study investigating the associations between sleep characteristics and CVD risk factors in adolescents living with T1D. The major strengths of the study include a well-characterized cohort of adolescents living with T1D and their peers living without diabetes, and the use of an objective and reliable method to assess the sleep characteristics over multiple days. However, we did not collect subjective sleep data, which may provide useful insights into group differences as to how those living with T1D perceive their own sleep quality. Furthermore, we could not perform subgroup analyses of continuous glucose monitoring and self-monitoring of blood glucose or continuous subcutaneous insulin infusion and multiple daily injections due to the small sample size of our study. Future research should assess the effects of technological advancements on sleep patterns and quality in people living with T1D. Additionally, the sample size in the peers without T1D group was less than the T1D group, and the power was based on the smaller sample. We also excluded diabetic individuals with complications. Furthermore, the cross-sectional nature of this study does not allow the determination of causality. Therefore, future research with a larger sample size is warranted. Moreover, an interventional study would allow for greater exploration of the potential mechanisms linking sleep and CVD risk factors in adolescents living with T1D. This study involved Chinese adolescents living with T1D; therefore, caution is needed when generalizing our study findings to other populations with different characteristics. In addition, potential confounders such as socioeconomic status of participants (education level of parents, health insurance, etc.) were not taken into consideration within our study.

## 5. Conclusions

In summary, adolescents living with and without T1D sleep less than the recommended 8 h per night. The association between sleep efficiency and LDL-C and triglycerides is independent of sleep duration, regardless of sex, age, and pubertal stage. Future research with a larger sample size is warranted to further explore sleep-promoting interventions on glycemic control and other CVD risk factors in adolescents living with T1D.

## Figures and Tables

**Table 1:** Comparison of descriptive data between adolescents with T1D and peers without diabetes.

	T1D (*n* = 48)		Peers without T1D (*n* = 19)		
	Mean	SD	Mean	SD	*p*
Sex (male/female) (%)	37.5/62.5		42.1/57.9		0.129
Pubertal stage (*n*)					0.543
Stage 1 (Not started)	18		7		
Stage 2 (Barely started)	8		1		
Stage 3 (Definitely underway)	12		6		
Stage 4 (Seems completed)	5		1		
Stage 5 (Completed)	5		4		
Ages (y)	14.02	2.89	13.58	3.46	0.601
Body mass (kg)	49.48	12.56	52.34	15.45	0.452
Height (m)	1.60	0.13	1.59	0.13	0.772
BMI (kg∙m^−2^)	18.97	3.10	20.38	3.32	0.119
Body fat (%)	29.28	9.54	28.42	6.61	0.765
Waist circumference (cm)	67.63	7.96	73.65	8.22	0.055
Systolic blood pressure (mmHg)	106.07	16.72	107.07	15.45	0.837
Diastolic blood pressure (mmHg)	65.24	11.65	65.75	9.88	0.878
Total cholesterol (mmol∙L^−1^)	4.03	0.81	3.14	0.67	0.001 *
HDL-C (mmol∙L^−1^)	1.48	0.28	1.29	0.42	0.078
LDL-C (mmol∙L^−1^)	2.31	0.72	1.74	0.38	0.005 *
Triglycerides (mmol∙L^−1^)	0.89	0.31	0.60	0.40	0.012 *
Diabetes duration (y)	3.64	2.29			
MDI (*n*)	30				
CSII (*n*)	18				
CGM (*n*)	26				
SMBG (*n*)	22				
HbA1c (%)	7.70	2.47			
Insulin dose (unit∙kg^−1^∙day^−1^)	0.87	0.29			

Data are presented as means and standard deviation (SD). T1D, type 1 diabetes; *n*, number; y, year; BMI, body mass index; HDL-C, high-density lipoprotein cholesterol; LDL-C, low-density lipoprotein cholesterol; VO_2_max, maximal aerobic power; METs, metabolic equivalent; CPM, counts-per-minute; * statistically significant difference (*p* < 0.05) between T1D and apparently healthy peers without diabetes; MDI, multiple daily injection; CSII, continuous subcutaneous insulin infusion; CGM, continuous glucose monitoring; SMBG, self-monitoring of blood glucose; HbA1c, glycosylated hemoglobin.

**Table 2:** Comparison of sleep characteristics between adolescents with T1D and peers without diabetes.

Sleep Parameters	T1D (*n* = 33) Mean (SD)	Peers without Diabetes (*n* = 16)	*p*-Value
Sleep-onset time (hh:mm) ^§^	22:51 (22:06–23:06)	22:42 (22:08–23:12)	0.932
Sleep-offset time (hh:mm) ^§^	6:54 (6:33–7:30)	6:54 (6:33–7:30)	0.757
Sleep latency (min) ^§^	1.78 (0.86–5.29)	2.17 (1.08–3.74)	0.662
Sleep efficiency (%)	86.30 (4.70)	86.43 (6.00)	0.933
Total sleep time (min)	421.30 (62.30)	432.02 (38.05)	0.534
Wake after sleep onset (min)	62.23 (24.10)	66.71 (32.49)	0.589
Number of awakenings (*n*)	20.82 (5.94)	21.21 (8.34)	0.848
Length of awakenings (min)	62.23 (24.10)	69.62 (39.64)	0.422

^§^ Data are presented as medians and 25–75th.

**Table 3:** Linear regression model examining the association between LDL-C and sleep parameters after adjusting for potential confounders in adolescents living without T1D.

	Dependent Variable: LDL-C					
Independent Variables	β	95% CI		*p*	Adjusted R^2^	R^2^
Sleep efficiency (%)						
Step 1 (unadjusted)	−0.0554	−0.07	−0.005	0.026	0.258	0.307
Step 2 (adjusted ^†^)	−0.04	−0.084	0.004	0.071	0.175	0.395
Total sleep time (min∙day^−1^)						
Step 1 (unadjusted)	−0.001	−0.003	0.001	0.201	0.027	0.065
Step 2 (adjusted ^†^)	−0.002	−0.003	0.000	0.060	0.383	0.597

^†^ Adjusted for age, sex, and pubertal stage.

**Table 4:** Linear regression model examining the association between LDL-C, triglycerides, and sleep efficiency after adjusting for potential confounders in the entire sample.

	Independent Variables: Sleep Efficiency					
Dependent Variable	β	95% CI		*p*	Adjusted R^2^	R^2^
LDL-C (mmol·L^−1^)						
Step 2 (adjusted)	−0.045	−0.082	−0.008	0.018	0.120	0.230
Triglycerides (mmol·L^−1^)						
Step 2 (adjusted ^††^)	−0.027	−0.048	−0.006	0.012	0.111	0.222

^††^ Adjusted for sleep duration, age, sex, diabetes, and pubertal stages.

## Data Availability

The datasets used and/or analyzed in this article are available from the corresponding author on reasonable request.
